# Authors’ Reply to: Interpretation Bias Toward the Positive Impacts of Digital Interventions in Health Care. Comment on “Value of the Electronic Medical Record for Hospital Care: Update From the Literature”

**DOI:** 10.2196/37419

**Published:** 2022-03-04

**Authors:** Jürgen Stausberg, Aykut Uslu

**Affiliations:** 1 Institute for Medical Informatics, Biometry and Epidemiology University Hospital Essen University Duisburg-Essen Essen Germany; 2 USLU Medizininformatik Düsseldorf Germany

**Keywords:** cost analysis, costs and cost analyses, economic advantage, electronic medical records, electronic records, health care, hospitals, computerized medical records systems, quality of health care, secondary data

We thank Shakibaei Bonakdeh [1] for the critical comment on the positive impact of digital interventions. It would be the ultimate success of medical informatics research to be recognized by hospital executives and managers. However, we are not convinced that the previous national initiatives for the implementation of electronic records were evidence-based, neither in England, Germany, nor the United States. Following the scene of electronic records for 30 years, recognizing the literature of 50 years, and having been responsible for the selection and management of electronic records in hospitals, our view was truthfully impartial. We were willing to accept scientific evidence independently of pre-existing opinions even if the clear result was a surprise [2].

First, we want to clarify that we did not evaluate digital interventions in general, as indicated by the letter’s headline. In our study, we sought to focus on electronic medical records (EMRs) as specific types of electronic records. However, we were confronted with several challenges concerning the definition and specification of the technology, as partly addressed in the letter. For example, excluding studies focusing on computerized physician order entry (CPOE) on the one hand did not mean excluding studies on EMRs that offer CPOE support on the other hand. It would be a step forward to have a standard not only for the reporting of results such as PRISMA (Preferred Reporting Items for Systematic Reviews and Meta-Analyses) but also for the labeling of digital interventions.

Concerning the results of the included studies, we aimed to draw the utmost benefits from the details. This could mean overriding the studies’ conclusions, as stated in our limitations. In the case of Adler-Milstein et al [3], we probably overweighted the improved efficiency in the post meaningful use period (2010/2011). We apologize if we did not meet the common appraisal in all cases. However, the results of Adler-Milstein et al [3] fully support the conclusions from the whole set of included studies with a clear positive effect on the quality of care and an ambiguous economical implication. The appropriate evaluation criteria of EMRs should be put up for discussion. It may be unreasonable to expect a reduction of mortality from its implementation. We would be honored if our series of reviews contribute to realistic expectations toward the effects of EMRs on different types of quality criteria.

Second, the other points of criticism were related to the context of EMR implementation. Studies about the effects of EMRs are faced with a double complexity [4]. The implementation of an EMR will involve the whole organization of hospital care, will alter health care processes, and will include a wide range of functionalities (EMR as a complex intervention). The setting of the implementation of an EMR is complex too, with different professions, different specialties, different levels of care, etc (complexity of the context). For example, the readiness for change and managing change might be more important for the success of an EMR implementation than specific technological issues [5]. It could be argued that well-prepared organizations will benefit more from an EMR than less prepared organizations. In extreme cases, less prepared organizations will get worse with the same EMR solution that helped well-prepared organizations to further improve patient outcomes. We agree with Shakibaei Bonakdeh [1] that there could be a coexistence effect in studies using secondary data. Hospitals attempting to improve care by implementing an EMR should be advised to analyze thoroughly their current state, to eliminate reasons for inappropriate care in advance, and to be well prepared for the technology.

Medical informatics science is confronted with the digitization of health care independently from its input and participation. Due to the high penetration of electronic records, interventional studies will no longer be possible for this technology in developed countries. Nevertheless, at times, society will seek help from medical informatics science, especially if political expectations fail. Frequently, low-hanging fruits determined the reaction of researchers in the past. It would be a major step forward if medical informatics science is willing to act as a collective community grounded on scientific evidence. With this regard, we express our appreciation for Shakibaei Bonakdeh’s [1] feedback.

## Abbreviations

CPOE: computerized physician order entry
EMR: electronic medical record
PRISMA: Preferred Reporting Items for Systematic Reviews and Meta-Analyses
